# Anti-Microbiological, Anti-Hyperglycemic and Anti-Obesity Potency of Natural Antioxidants in Fruit Fractions of Saskatoon Berry

**DOI:** 10.3390/antiox8090397

**Published:** 2019-09-13

**Authors:** Sabina Lachowicz, Rafał Wiśniewski, Ireneusz Ochmian, Katarzyna Drzymała, Stanisław Pluta

**Affiliations:** 1 Department of Fermentation and Cereals Technology, Wrocław University of Environmental and Life Science, 37, Chełmońskiego Street, 51-630 Wroclaw, Poland; 2 Department of Food Technology and Human Nutrition, Faculty of Biology and Agriculture, University of Rzeszów, Zelwerowicza 4 St., 35-601 Rzeszów, Poland; rwisniewski@ur.edu.pl; 3 Departament of Pomology, West Pomeranian University of Technology, J. Słowackiego 17 Str, 71-434 Szczecin, Poland; ireneusz.ochmian@zut.edu.pl; 4 Department of Biotechnology and Food Microbiology, Wrocław University of Environmental and Life Science, 37, Chełmońskiego Street, 51-630 Wroclaw, Poland; katarzyna.drzymala@up.wroc.pl; 5 Research Institute of Horticulture, Department of Horticultural Crop Breeding, Konstytucji 3 Maja 1/3, 96-100 Skierniewice, Poland; Stanislaw.pluta@inhort.pl

**Keywords:** *Amelanchier alnifolia*, biological activity, polyphenol compounds, free amino acids, anti-diabetic, in vitro, PCA

## Abstract

The aim of the present work was to evaluate for content of phytochemicals (monophosphate nucleotides, free amino acids, polyphenols), and for anti-microbiological, anti-diabetic (ability to inhibit pancreatic lipase, α-glucosidase, and α-amylase), and antioxidant activities in seven selected fruit and fruit fractions of *Amelanchier alnifolia*. Most of the fruit and fruit fractions analyzed in this study have not been examined in this respect until now. The content of monophosphate nucleotides and free amino acids were tested by ultra-performance liquid chromatography coupled with photodiode array detector and electrospray ionization-mass spectrometry (UPLC-PDA-ESI-MS). The distribution of the examined compounds and biological activity differed significantly depending on the tested fruit and parts of the fruit. Cultivars “Smoky” and “Thiessen” had a high content of essential free amino acids, monophosphate nucleotides, and the highest antioxidant activity. They were also accountable for the high ability to inhibit *Enterococcus hirae* (anti-bacterial activity), of activity toward α-amylase, α-glucosidase, and pancreatic lipase. Moreover, the fruit peel was abundant in polyphenolic compounds and showed the highest antioxidative activity, which were strongly correlated with each other. In addition, the peel was characterized by a high concentration of monophosphate nucleotides, free amino acids, and were responsible above all for the strong ability to inhibit pancreatic lipase enzymes contributing to the development of obesity. The seeds were rich in uridine 5’-monophosphate, and total essential and non-essential free amino acids, whose contents correlated with the inhibitory activity toward α-amylase and α-glucosidase. The fruit flesh showed a high content of total free amino acids (hydroxy-L-proline, *O*-phosphoethanolamine, L-citruline). There was a positive correlation between antioxidant capacity and the content of polyphenolic compounds, nucleotide, and ability to inhibit pancreatic lipase, and between anti-hyperglycemic and free amino acids in fruits and fruit fractions. Therefore, the tested fruit of *A. alnifolia* and their fractions could be essential ingredients of new functional products and/or probiotic food.

## 1. Introduction

Fruits and vegetables are valuable sources of not only carbohydrates, proteins, minerals, pectins, and organic acids, but also of bioactive secondary metabolites such as natural antioxidants. Polyphenolic compounds including mainly anthocyanins, phenolic acids, and polymeric proanthocyanins are particularly noteworthy and they correlate positively with antioxidant activity. They exhibit primarily the sensory properties of food and exhibit anticarcinogenic, antiulcer, cardio protective, and antidiabetic effects as well as inhibit the growth and development of bacteria [1,2]. These metabolites constitute a numerous group of health-promoting compounds which are unevenly distributed in various parts of plants [3]. The peel of berries is an excellent source of flavanols and anthocyanins, while the fruit flesh contains mainly flavonols. In turn, proanthocyanidins and phenolic acids prevail in leaves [4].

Fruits are rich in other compounds showing a number of health-promoting benefits such as, for example, amino acids and nucleotides. Amino acids play a key role in the organism. They are also responsible for the hormonal balance of our cells and DNA. Amino acids produce proteins, and participate in the production of, among others, insulin, adrenaline, body fluids, and neurotransmitters [5,6], and contribute to an amelioration in immune response, impact on fatty acids, and impact on healing of gastrointestinal tract damage [7]. Monophosphate nucleotides are the basic components of nucleic acids (DNA and RNA). In the cell they play a significant role in the processes of metabolism and signal transmission [5,6]. Nucleic acids are present in raw and processed food consumed by humans, especially in vegetables, fruits, fish, seafood, and meat. The presence of nucleic acids has an impact on the sensory properties of the food consumed, mainly due to the incidence of guanosine monophosphate (GMP nucleotide) which is considered to be an umami flavor enhancer [8]. These nucleotides play a role in the inflammatory process and apoptosis. These compounds are tested for their use as potential anti-inflammatory, analgesic, and treatment for cardiovascular and neurodegenerative diseases [8,9,10].

The incidence of type 2 diabetes is directly proportional to the occurrence of obesity among the human population, which is caused by high intakes of carbohydrates. In the human body, carbohydrates are degraded by intestinal α-glucosidase and pancreatic α-amylase, which hydrolyze disaccharides and oligosaccharides into monosaccharides that are ready for absorption. In addition, it has been proved that polyphenolic compounds exhibit anti-hypoglycemic effects due to their antioxidative potential. Therefore, they are used in the prevention of noninsulin-dependent diabetes, because they inhibit the action of α-amylase and α-glucosidase, slowing down glucose absorption into the bloodstream [11,12,13].

It is known that pancreatic lipase is the major enzyme responsible for dietary fat absorption, as it hydrolyzes triglycerides from food into free fatty acids and monoacylglycerides. Inhibition of its activity reduces the absorption of dietary fat and can lead to a beneficial effect of weight loss. Therefore, the use of fruit extracts rich in phytochemical compounds as inhibitors of this enzyme can be an alternative approach to overweight and obesity treatment [12]. 

Nowadays, special attention is paid to foods with health-promoting properties that contain no synthetic additives of antioxidants such as butylated hydroxytoluene (BHT) [14,15]. However, in the food processing industry there is still necessity to use antioxidant substances to prolong storage stability of foods. Therefore, by being a good source of these properties, pomace may be used as a food additive. In order to meet today’s requirements of global consumers as well as food producers it seems necessary to study and promote raw materials and their components which have a high nutritional value and exert a beneficial impact on human health. The fruit of the Saskatoon berry (*Amelanchier alnifolia*) seems to be a valuable raw material as a potentially rich source of phytochemicals and antioxidant properties. However, little is known about the profile of monophosphate nucleotides, free amino acids and their exact distribution, and biological properties of particular parts of the fruit of this species. Therefore, the objective of this study was to evaluate the content of phytochemicals (monophosphate nucleotides, free amino acids, and polyphenolics), and for anti-diabetic (hyperglycemia—α-glucosidase, α-amylase and obesity—pancreatic lipase), antioxidant capacity (oxygen radical absorbance capacity (ORAC) assay), and anti-microbiological properties of the fruit and their peel, flesh and seeds of seven selected *A. alnifolia*. In addition, correlations were determined between phytochemicals content in the fruit of the tested genotypes and their fractions and important variables of the antidiabetic, antimicrobial, and antioxidant effects.

## 2. Materials and Methods 

### 2.1. Plant Materials 

The fruit and their fractions, such as peel, flesh, and seeds of seven selected Saskatoon berry genotypes were used in these studies. The research material contained “Martin”, “Pembina”, “Smoky”, and “Thiessen” cultivars (cvs.) and “no. 5/6”, “type N”, and “type S” clones [16,17]. The fruit samples were collected from Skierniewice, Poland (51°55′24″ N, 020°5′58″ E). The raw material was directly frozen in liquid nitrogen and freeze-dried (24 h; Christ Alpha 1–4 LSC; Germany). The homogeneous dry material was obtained by crushing the dried tissues using a closed laboratory mill (IKA A.11, Germany). The powders were kept in a refrigerator (–80 °C) until extract preparation.

### 2.2. Determination of Color

The pigment measurement (color) of the fruit and their fractions of the Saskatoon berry was analyzed in a transmitted mode evaluated by photocolorimetric method tested in CIE L*a*b* system, as described by Ochmian et al. [18]. The a* value showed the place of appearing in the color gamut, in the range from green to red on the surface of dried fruits of analyzed genotypes. The b* parameter described the color in the range from yellow to blue on the surface of dried fruits of tested genotypes. The value of L* parameter ranges from 0 to 100, black to white, respectively. Relative changes in anthocyanins, as a normalized anthocyanin index—NAI = (I780 − I570)/(I780 + I570) with a disposition of both parameters normalized to between –1 (redness) and +1 (red) was measured [18].

### 2.3. Determination of Monophosphate Nucleotides

The qualitative analysis of monophosphate nucleotides was performed as reported by Yamaoka et al. [19]. LC–MS was performed under the following conditions: instrument, Waters UPLC™ and system with an electrospray ionization (ESI) interface and a quadrupole mass detection system (Waters, Milford, MA, USA); software, MassLynx^TM^; column, ACQUITY UPLC™ Column HSS T3 (2.1 mm ID and 100 mm length, 1.8-μm particle size) (Waters, Milford, MA, USA). The ESI source was operated at 120 °C with a desolvation temperature of 450 °C, 800 L/h desolvation gas flow rate, and a capillary voltage set at 3.5 kV. The cone voltage was 30 V and collision energies 30 eV. Integration and quantitation were performed using the Waters MassLynx^TM^ software. Detection of these compounds was performed in the negative ionization. Monophosphates nucleotides (xanthosine 5’-monophosphate (XMP), cytidine 5’-monophosphate (CMP), uridine-5’-monophosphate (UMP), thymidin-5’-monophosphate (TMP), adenosine-5’-monophosphate (AMP), inosine 5’-monophosphate (IMP), guanosine-5’-monophosphate (GMP)), were purchased from Sigma–Aldrich (Wrocław, Poland). All data were obtained in triplicate. The results were expressed as mg/100 g of dry weight (d.w.).

### 2.4. Determination of Free Amino Acids

Milled freeze-dried samples were used for free amino acid determination. Ultra-performance liquid chromatography coupled with photodiode array detector and electrospray ionization-mass spectrometry (UPLC-PDA-ESI-MS) analysis was carried out on a Waters ACQUITY (Waters Corporation, Milford, U.S.A.). We used parameters of detection as detailed by Roucher et al. [20] and Armenta et al. [21]. The ESI source was operated at 120 °C with a desolvation temperature of 450 °C, 800 L/h desolvation gas flow rate, and a capillary voltage set at 3.5 kV. The cone voltage varied from 20–35 V and collision energies within 10 to 40 eV, depending on the free amino acid investigated. Integration and quantitation were performed using the Waters MassLynx^TM^ software. Detection of these compounds was performed in the positive ionization. The qualitative analysis was previously described by Roucher et al. [20]. Amino acid standards (certified reference material) was purchased from Sigma–Aldrich (Wrocław, Poland). All data were obtained in triplicate. The results were expressed as mg/100 g of d.w.

### 2.5. Determination of Polyphenolic Compounds

The analysis of polyphenolic compounds were determined by the Folin–Ciocalteu method [22]. An aliquot (100 μL) of extract was mixed with 2000 μL of distilled water and 200 μL of Folin–Ciocalteu phenol reagent. Two hundred microliters of sodium carbonate solution (200 g/L) was added to the mixture. The mixture was incubated at 20 °C for 1 h in darkness. The absorbance was read at 765 nm on a UV-VIS spectrophotometer (Shimadzu UV-2401 PC, Kyoto, Japan). Solutions of gallic acid from 0 to 500 mg/L were measured with the same procedure, for the creation of the calibration curve. All data were obtained in triplicate. The results were expressed as g GAE/100 g of d.w.

### 2.6. Determination of Anti-Microbiological Potency

The antimicrobial activity of the freeze-dried fruit tissue was tested on the following strains of microorganisms: (1) Gram positive bacteria *Staphylococcus aureus* (ATCC 9538), *Bacillus subtilis* (ATCC 6633), *Enterococcus hirae* (ATCC 10542), and *Enterococcus faecalis* (ATCC 29212); (2) Gram negative bacteria *Escherichia coli* (ATCC 10536), *Pseudomonas aeruginosa* (ATCC 15442), *Vibrio harveyi* (ATCC 12126), and *Proteus mirabilis* (ATCC 2011); and (3) yeast *Candida albicans* (ATCC 10231). All microorganisms were obtained from the culture collection of the Department of Biotechnology and Food Microbiology (Wrocław). The bacteria were grown in nutrient broth medium at 37 °C, except *B. subtilis* (ATCC 6633) which was grown at 30 °C. The yeast was grown in Yeast Extract Peptone Glucose (YPD) medium at 30 °C. The agar was added to the medium at a concentration of 2% when it was needed.

### 2.7. Determination of Enzyme Inhibition Potency

Anti-diabetic activity, α-amylase, α-glucosidase inhibitory, and lipase activity effect of the materials were described previously by Nakai et al. [23], Podsędek et al. [12], and Nickavar et al. [24]. The extraction of mixed parts of fruits was done with 70% acetone (or water) at room temperature for 60 min with constant stirring. After centrifuging at 4000 rpm for 10 min, and filtration, the supernatants were concentrated at 40 °C (vacuum evaporator) to remove the acetone and the aqueous phase was diluted with water. For further analytical and biological activity assays, a gradient of concentrations was prepared via serial dilution of the fruit extracts in pure water. The amount of the inhibitor (expressed as mg of fruit per 1 mL of reaction mixture under assay conditions) required to inhibit 50% of the enzyme activity was defined as the IC_50_ value. The IC_50_ of the fruits tested was obtained from the line of the plot of the fruit concentration in 1 mL of reaction mixture versus the % inhibition. All samples were assayed in triplicate.

### 2.8. Determination of Antioxidant Activity

The oxygen radical absorbance capacity (ORAC) method was applied in our studies as reported by Kapusta et al. [25]. The antioxidants were presented as mmol vit. C/100 g d.w.

### 2.9. Statistical Analysis

Statistical analysis, including one-way ANOVA and the principal components analysis (PCA) were conducted using Statistica 12.5 (StatSoft, Kraków, Poland). Significant differences (*p* ≤ 0.05) between mean were assessed by Duncan’s *t*-test.

## 3. Results and Discussion

### 3.1. Determination of Color

The appearance and color of fruit tested in this study are very important stimuli for the receptors, and thus, play an important role in consumer’s determination of food quality. Fruit color can encourage consumption by influencing the suggestions of certain flavors and/or discourage by warning of their rottenness [26,27]. Results of color determination of the dried fruit and their parts of the Saskatoon berry analyzed in the transmitted mode with the photocolorimetric method in the CIE L*a*b* system are presented in Table 1. The value of a* parameter (color range from green to red) determined on the surface of dried fruit of the tested fruit ranged from 18.33 (“clone no 5/6”) to 34.55 (“Smoky”). It strongly depended on the fruit and parts of fruit. Regarding the tested fruit parts, the value of a* parameter evaluated for the peel was significantly higher compared to flesh and seeds. For the tested fruit, it was on average 38.20, 12.15 and 9.47, respectively (Table 1). These results were in agreement with the normalized anthocyanin index (NAI) (meaning −1 and +1, redness and red, respectively). While, the anthocyanin dyes were responsible for the color of fruit of the Saskatoon berry fruits, the NAI showed that the highest content of anthocyanins was noted in the fruit of cvs. “Smoky” and “Thiessen” (0.71), while the lowest one was in “clone type N” (0.57). In addition, the fruit peel was characterized by the highest NAI, reaching 0.85 on average. It was confirmed that the peel was red and contained about twice more anthocyanins compared to the flesh. According to literature, the value of a* parameter measured in the chokeberry fruit is similar, which is confirmed in the reports by Horszwald et al. [28] and Lachowicz et al. [29].

The color of the surface of the dried fruit of the tested fruit determined by the b* parameter (from yellow to blue colors) ranged from 18.10 (“clone no 5/6”) to 23.03 (“Smoky”). The highest value of the b* parameter (36.29) was analyzed in the fruit flesh, whereas an average value of this parameter determined in the fruit peel and seeds was lower (1.4 and 1.6 times, respectively) (Table 1). It was shown that cvs. “Smoky” and “Thiessen” contained the highest quantity red-coloring substances and a smaller quantity of yellow-coloring ones, and that the flesh of all fruit was characterized by a higher contribution of yellow and a smaller contribution of red color.

The value of L* parameter (reaching from 0 to 100, black to white, respectively) ranged from 32.71 (“Thiessen”) to 49.31 (“clone no 5/6”), and this parameter is usually used for tracking color changes [18]. While it was on average 54.14 in the fruit flesh and was 2.5 higher compared to the peel., “Smoky” and “Thiessen” had the darkest fruit, which undoubtedly could affect obtaining a light juice and dark pomace. Similar results and observation were obtained in the case of Saskatoon berry cultivars [28]. However, blueberry fruits are on average 1.4 times brighter compared to the Saskatoon berry [30].

### 3.2. Determination of Monophosphate Nucleotides 

Results of the qualitative and quantitative analysis of monophosphate nucleotides in the fruit of the investigated Saskatoon berry cultivars and breeding clones and their parts are presented in Table 2. The literature data show that monophosphate nucleotides have not been analyzed in the fruit and their components of Saskatoon berry. In our study, it was found that among the seven forms of monophosphate nucleotides identified in the analyzed fruit samples the major ones were as follows: xanthosine 5′-monophosphate (XMP; 30%–35%) > cytidine 5’-monophosphate (CMP; 27%–31%) > uridine 5’-monophosphate (UMP; 16%–25%) ≥ thymidine 5’-monophosphate (TMP; 5%–15%) > adenosine 5’-monophosphate (AMP; 4%–8%) > inosine 5′-monophosphate (IMP; < 3%) ≥ guanosine 5’-monophosphate (GMP; < 3%). The total content of monophosphate nucleotides in fruit ranged from 24.59 mg/100 g d.w. (“clone type N”) to 34.75 mg/100 g d.w. (cv. “Pembina”). The predominant monophosphate nucleotides were XMP and CMP with average contents at 11.88 and 10.56 mg/100 g d.w., respectively. Considering particular parts of the fruit of the tested genotypes, the peel had the highest content of monophosphate nucleotides, which was 2.1 and 1.6 times higher than in the flesh and seeds. The exception was CMP and XMP, whose contents fruit flesh were 1.2 and 1.3 times higher than in the peel. According to literature, GMP, CMP, UMP, and AMP were identified among monophosphate nucleotides in dried jujube (*Ziziphus* jujube) fruits. The content of these compounds in jujube fruits is similar [31]. In turn, AMP content in banana (*Musa* L.) peel and lychee (Litchi *chinensis*) pericarp tissue is 1.3 times lower, compared to the peel of both analyzed fruit species [32]. Furthermore, monophosphate nucleotides enhance the palatable or umami taste in order GMP > IMP > XMP > AMP, and also work with free amino acids, with the impact of intensifying the flavor sensation. This happens because they bind as glutamate to the same receptor (T1R1 + T1R3) [7,33]. Hence the delicious, sweet taste, or perception of satisfaction the consumption of the Saskatoon berry fruit may contribute. Therefore, the analyzed fruit and their fractions of *A. alnifolia* could be excellent ingredients of new functional products. 

### 3.3. Determination of Free Amino Acids

In our study, the qualitative and quantitative analysis of free amino acids were investigated in the fruit of seven selected fruit and their fractions, and respective results are presented in Table 3 and Table 4. In total, 23 compounds were identified, including 11 essential and 12 non-essential free amino acids. Among the 12 non-essential free amino acids, the major ones were: arginine (~44.4%) > phenylalanine (~27.2%) > histidine (~10.1%) > tryptophan (~6.3%) > *O*-phosphoethanolamine (~3.9%) > lysine (~2.8%) > leucine (~2.1%) > valine (~1.5%) > 3-methyl-L-histidine (~1.1%) ≥ methionine, threonine, urea (< 1.0%). The major ones among 11 essential free amino acids were asparagine (~40.2%) > aspartic acid (~28.0%) > glutamic acid (~13.1%) > serine (~10.2%) > proline (~4.8%) > L-ornithine (~1.5%) > tyrosine (~1.4%) > alanine, L-citruline, hydroxy-L-proline, and glycine (< 1.0%).

As it was reported by Mazza [34], 16 total amino acids from protein including nine essential and seven non-essential ones were identified in the fruit of the Saskatoon berry in Canada. The following free amino acids: *O*-phosphoethanolamine, 3-methyl-L-histidine, urea, asparagine, L-ornithine, L-citruline, and hydroxy-L-proline have not been identified in these fruits until now. The average content of free amino acids in the fruit of analyzed genotypes was 46.58 mg/100 g d.w. and ranged between 34.71 mg/100 g d.w. (“clone no 5/6”) and 62.44 mg/100 g d.w. (“Martin”). Our results differed significantly from those obtained in Canada, while the average content of total amino acids from protein was sixteen times higher than in the content of free amino acids in the fruits [34]. In turn, in the immature, mid ripe and ripe medlar (*Mespilus germanica* L.) fruit the average content of total amino acids from protein is significantly ten times higher [35]. Additionally, asparagine is also a predominant amino acid in medlar fruit. This amino acid is used in the treatment of anti-arthritis and in the form of mercury salt against keels [35]. In turn, 21 free amino acids were identified in quince (*Cydonia oblonga*) seeds and their content is on average four times lower than in the seeds of the Saskatoon berry [36]. According to Zhang et al. [37], the differences in the content of free amino acids in fruits depend mainly on fruit maturity, growing conditions, part of the plant, genotype, and analytical methods. Moreover, glutamic and aspartic acids identified in fruit and fruit fractions are monosodium glutamate-like components, and give the typical taste of food, the umami or palatable taste. Besides, the umami flavor is the typical taste of monosodium glutamate, and also monophosphate nucleotides [7,33]. The average content of this free amino acid in fruit of Saskatoon berry was 1.1 and 1.3 times higher compared to the flesh, the seeds, and the peel, which was on average five times lower to content of monosodium glutamate-like compounds in mushrooms (from 60 to 4420 mg/100 g d.w.) [7]. What is more, this content was in the low range (<500 mg/100 g) of this component according to the division described by Yang et al. [38]. 

### 3.4. Determination of Polyphenolic Compounds

The total average content of polyphenols was 1.79 g GAE/100 g d.w. in fruit of the tested Saskatoon berry fruits and 0.98 g GAE/100 g d.w. in fruit components (Table 5). The polyphenols in berries of the analyzed fruit ranged from 1.11 g GAE/100 g d.w. (“clone no 5/6”) to 2.27 g GAE/100 g d.w. (“clone type N”). Similar results were obtained by Rop et al. [39] in the Czech Republic and by de Souza et al. [14] in Canada. According to Lavola et al. [40] and Lachowicz et al. [16,17], the average content of polyphenols in fruit of seven tested Saskatoon berry cultivars and clones was twice higher than the results mentioned above. It is known that significant differences in contents of polyphenolic compounds in fruits of the same fruit collected in different years might mainly be due to the analytical method used for their determination. In our study, the highest average polyphenols content was obtained in the fruit peel (2.00 g GAE/100 g d.w.), while the lowest one in seeds (0.32 g GAE/100 g d.w.). A similar trend in the distribution of polyphenols is observed by Xu et al. [41] in different grape cvs. grown in China. However, Pantelić et al. [42] state an opposite tendency in red grapes from Serbia; in which the highest amount of the polyphenols was found in: seeds > skin > pulp. Results of our study showed that the polyphenols content in the fruit of the tested berry and their parts depended significantly (*p* ≤ 0.05) on the analyzed fruit fraction, i.e., peel, flesh, and seeds, and on cultivar/genotype. In turn, the average polyphenols are similar in seeds of purple, white, and red grapes from China, whereas in the skin of white, purple, and red grapes the polyphenols are 3.0, 1.7, and 1.3 times lower compared to the peel of the analyzed grape genotypes [41]. The amount of bioactive compounds in the peel, the flesh, and the seeds of miracle berry (*Synsepalum dulcificum*) is 1.2, 1.8, and 3.5 times higher than in the fruit fractions of *A. alnifolia* [43]. 

### 3.5. Determination of Antibacterial Properties

Results of analyses of the microbiological immunity of Saskatoon berry against such microorganisms as Gram-positive bacteria *Staphylococcus aureus* (ATCC 9538), *Bacillus subtilis* (ATCC 6633), *Enterococcus hirae* (ATCC 10542), and *Enterococcus faecalis* (ATCC 29212), Gram-negative bacteria: *Escherichia coli* (ATCC 10536), *Pseudomonas aeruginosa* (ATCC 15442), *Vibrio harveyi* (ATCC 12126), and *Proteus mirabilis* (ATCC 2011), and on yeast *Candida albicans* (ATCC 10231) are presented in the Table 5. Unlike the extracts of the analyzed fruit parts, the extracts of whole fruits showed resistance to the growth and development of only one strain of bacteria—*Enterococcus hirae* (ATCC 10542). This bacteria belongs to the *Enterococcaceae* family, which is a natural flora found primarily in the intestines of humans and animals, but also in the mouth, genitals, and skin. It is also often detected in water, sewage, and food. It is mainly responsible for the inflammation of the mucous membrane of the rectum. The fruit extracts of the analyzed fruits had strong effect on growth inhibition of *Enterococcus hirae,* especially the extract of “clone type S” fruit, but also extracts of cvs. “Smoky”, “Thiessen”, and “clone type N” showed relatively high anti-bacterial activity. The lowest inhibition of this bacterium growth was demonstrated for cv. “Pembina” and “clone no 5/6”. The high growth inhibition of this microorganism was associated with the high content of polyphenolic compounds, nucleotides, and free amino acids (nonessential) in the fruit samples. It was confirmed by a strong positive correlation (*r*^2^ = 0.525, 0.696, and 0.651, respectively) between variables. Such findings were also confirmed by other authors [44,45]. While, Tian et al. [45] show strong antibacterial activities of the leaf and branch extracts of the Saskatoon berry against *Escherichia coli* (VTT E-94564), *Staphylococcus aureus* (VTT E-70045), *Listeria monocytogenes* (VTT E-97783), and *Bacillus cereus* (VTT E-93143). Moreover, the Saskatoon berry fruit showed antibacterial properties against *E. coli* (VTT E-94564) and *Staphylococcus aureus* (VTT E-70045).

### 3.6. Determination of Antioxidant Activity

Results of determinations of the antioxidant properties of the seven selected fruit using in vitro ORAC tests are presented in Table 5. Significant differences of the antioxidant capacity were determined in the fruit of the tested fruit and their fractions (peel, flesh, and seeds). The antioxidative activity measured with the ORAC test ranged from 35.37 mmol vit. C/100 g d.w. (“Martin”) to 62.05 mmol vit. C/100 g d.w. (“Smoky”). Our results determined for the Saskatoon berry fruit were similar to those reported by other authors [16,17,39]. The highest antioxidant potential measured with the ORAC assay was determined in fruit peel (average 122.65 mmol vit. C/100 g d.w.), while the lowest one was in seeds (average 11.79 mmol vit. C/100 g d.w.). Our findings were in an agreement with results reported by other authors: Xu et al. [41] and Yilmaz et al. [46]. In turn, the antioxidant potential in the analyzed seeds of purple, white, and red grape cultivars grown in China [41] is 3.0, 2.0, and 1.8 times lower compared to the seeds of fruit investigated in our study. As it was presented by Xu et al. [41], the antioxidant activity in measured the grape fruit-peel was similar to our results. According to Yilmaz et al. [46], differences in the antioxidant properties of various fruit parts depended mainly on fruit maturity, growing conditions, part of plant, and genotype. 

It is known that the antioxidative potential depends on the content of bioactive components, especially polyphenolics. In our study on the Saskatoon berry, a strong Pearson’s correlation was determined between the content of polyphenols and antioxidant capacity (*r*^2^ = 0.983, ORAC assay), and between anthocyanins and antioxidants (*r*^2^ = 0.818, ORAC assay) [16]. A similarly high correlation between the above-mentioned variables was previously presented by Rop et al. [39] in Czech Republic, and also by Hu et al. [47] from Canada, who show a high antioxidant potential of two cvs. “Thiessen” and “Smoky”. Additionally, the Saskatoon berry fruits might exert an intracellular antioxidative effect. Results obtained by Wang and Mazza [48] show that the extracts rich in polyphenols of these fruits could inhibit the production of nitric oxide (NO). That activity was closely correlated with the content of polyphenolic components in the Saskatoon berry fruits. Our findings confirmed that the antioxidative potential of the tested fruits was reinforced by nucleotides (*r*^2^ = 0.428, ORAC assay).

### 3.7. Determination of Enzyme Inhibition Properties 

The biological activity (as IC_50_) of the fruit of seven Saskatoon berry fruits and their fractions was evaluated by the anti-diabetic (hyperglycemia—α-glucosidase, α-amylase; and obesity—pancreatic lipase) assay (Table 5). Significant differences (*p* < 0.05) were noted in the inhibitory activities of α-glucosidase and α-amylase in the tested Saskatoon berry fruits and their parts. The inhibition of α-amylase in the analyzed fruit ranged from 18.33 (“clone type N”) to 31.70 IC_50_ (“Smoky”). While, the inhibition of α-glucosidase in these fruit samples was between 27.83 (“Martin”) and 42.23 IC_50_ (“Smoky”). Contents of polymeric procyanidins and free amino acids (essential and nonessential) were strongly correlated with the inhibitory activity toward α-amylase (*r*^2^ = 0.146, 0.685, and 0.507, respectively) and α-glucosidase (*r*^2^ = 0.586, 0.939, and 0.536, respectively). According to Burns Kraft et al. [49] the fruit extract of the Saskatoon berry inhibits enzymes responsible for diabetes and also affects glucose uptake by the insulin-like effect. Additionally, an extract from these fruit improves glycogen accumulation in the basal state. A similar inhibition of α-amylase was found in the bilberry (*Vaccinium myrtillus)*, blackberry (*Rubus fruticosus)*, pink grape (*Vitis vinifera*), lingonberry (*Vaccinium vitis-idaea)*, and pomegranate (*Punica granatum*), while toward α-glucosidase inhibition found in the blue honeysuckle (*Lonicera caerulea*), and blueberry (*Vaccinium corymbosum*) [12]. The peel fruit extracts were the most effective α-glucosidase and α-amylase inhibitors. However, the inhibitory effect obtained was moderate for α-amylase IC_50_ 14.00 and α-glucosidase IC_50_ 28.88, and was more effective than in the flesh and seeds (2.3 and 1.2 times, and 3.1 and 2.5 times, respectively). In peel of the tested Saskatoon berry fruit, the inhibition of α-amylase significantly higher than the activity of α-glucosidase. Moreover, high rates of inhibition of both α-amylase and α-glucosidase could contribute to the malfunction of the gastrointestinal tract by disrupting bacterial fermentation in the colon due to residues of undigested carbohydrates. For this reason, stronger inhibition of only one enzyme is desired [13,50]. However, a more effective inhibition effect is seen in chokeberry (*Aronia melanocarpa*), blackcurrant (*Ribes nigrum* L.), red currant (*Ribes spicatum* Robson), and green and red gooseberry (*Ribes grossularia*) [12]. 

Significant differences (*p* < 0.05) concerning the inhibitory activity towards pancreatic lipase were noted between the fruit and of the tested Saskatoon berry fruit and their parts (Table 5). Our findings showed no inhibitory activity toward this enzyme in the seeds. However, the inhibitory effects in the tested fruit ranged from 81.93 IC_50_ (“clone type N”) to 132.63 IC_50_ (“Smoky”). A higher (1.3 times) inhibitory activity of pancreatic lipase was obtained in the fruit peel than in the flesh. The inhibitory influence toward pancreatic lipase was stimulated by the polyphenols and nucleotides, resulting in high correlation coefficients (*r*^2^ = 0.625 and *r*^2^ = 0.899, respectively), and also by anthocyanins and phenolic acids (*r*^2^ = 0.683 and 0.822, respectively) [16]. As reported by Podsędek et al. [12], a similar, high inhibitory effect on pancreatic lipase was determined only in the Asian pear cv. “Nashi”, but its low activities were also shown in fruits of bilberry (*Vaccinium myrtillus*), blackberry (*Rubus fruticosus*), chokeberry (*Aronia melanocarpa*), blueberry (*Vaccinium corymbosum*), and lingonberry (*Vaccinium vitis-idaea*).

### 3.8. Principal Components Analysis (PCA)

**Figure 1:**
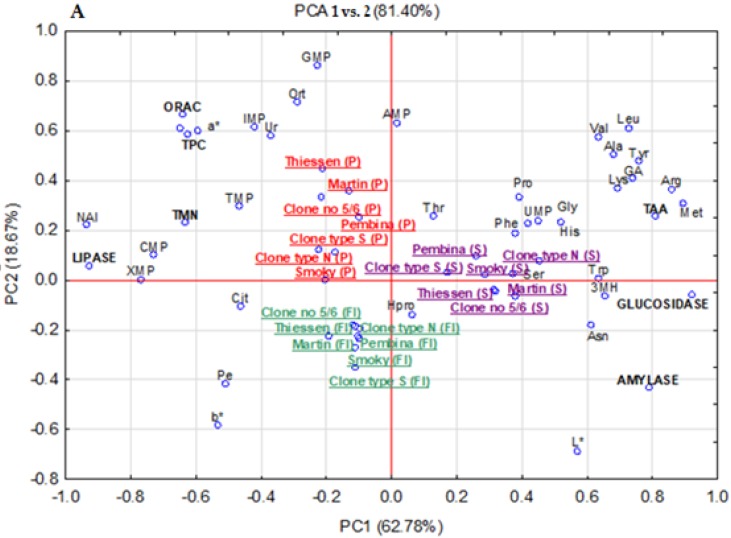

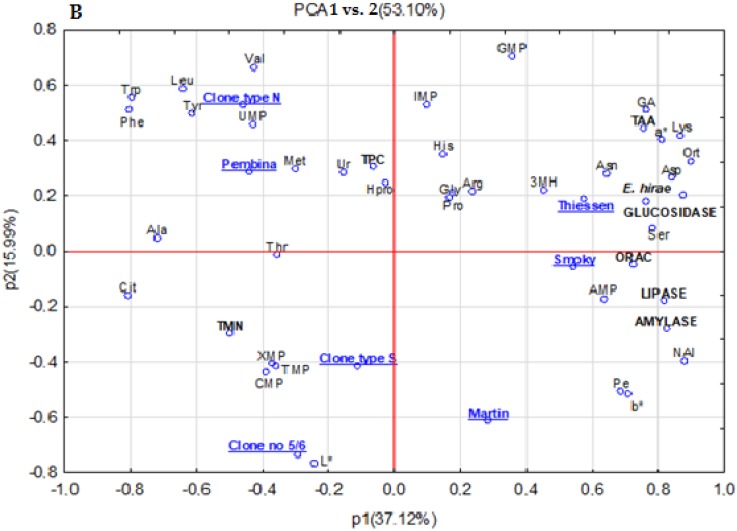
The principle component analysis (PCA) for fruit fractions, as seeds (S), peel (P), and flesh (F) (**A**) and the genotypes (**B**) Explanation: EnTAA, total of endogenous free amino acids; EgTAA, total of exogenous free amino acids; TMN, total of monophosphate nucleotides; PC, sum of phenolic compounds; IMP, inosine 5′-monophosphate, UMP, uridine-5’-monophosphate, CMP, cytidine 5’-monophosphate, GMP, guanosine 5’-monophosphate, XMP, xanthosine 5′-monophosphate, TMP, thymidine 5’-monophosphate, AMP, adenosine 5’-monophosphate. Arg, arginine; His, histidine, Met, methionine; Lys, lysine; Leu, leucine; Thr, threonine; Val, valine; Phe, phenyloalanine; PE, *O*-phosphoethanolamine; Try; tryptophan; Ur, urea; 3MH, 3 methyl-L-histidine. Glu, glutamic acid; Asp, aspartic acid; Pro, proline; Hpro, hydroxy-L-proline, Ser, serine; Gly, glycine; Ala, alanine; Tyr, tyrosine; Cit, L-citruline; Ort, L-ornithine; Asn, asparagine.

Results of the PCA concerning the identification, concentration, and distribution of the analyzed compounds in the fruit and their fractions are depicted in Figure 1. The PCA for fruit peel, flesh, and seeds of fruit of seven fruit explained 81.40% of total variance, where PC1 accounted for 62.78% and PC2 for 18.67% (Figure 1A). The statistical method pointed out three major parts. The first part included fruit peel which was abundant in the polyphenols and showed the highest antioxidative activity, with polyphenols content and oxidative activity being strongly correlated with each other. Moreover, fruit skin was characterized by a high concentration and distribution of total monophosphate nucleotides, and their individual components as AMP, XMP, IMP, GMP, TMP, free amino acids as urea, L-ornithine, the value of a* coefficient and NAI, which was indicative of the high contribution of the red pigment, and was also responsible above all for the inhibition of the activity of pancreatic lipase implicated in obesity development. The next part contained seeds rich in the uridine 5’-monophosphate, and the sum of exogenous and endogenous free amino acids, and their individual compounds as arginine, histidine, methionine, lysine, leucine, threonine, valine, phenyloalanine, tryptophan, 3 methyl-L-histidine, glutamic acid, aspartic acid, proline, serine, glycine, alanine, tyrosine, and asparagine, contents of which were correlated with results of the antioxidant assay. In addition, the seeds were responsible above all for the inhibition of activities of α-amylase and α-glucosidase enzymes that contribute to hyperglycemia development. The last part, i.e., fruit flesh, depicted the high content of free amino acids as hydroxy-L-proline, *O*-phosphoethanolamine, L-citruline, and parameter L*, and b*. Generally, the distribution of the examined compounds and biological activity differed significantly depending on the fruit part tested.

Furthermore, the PCA graph plotted for the seven analyzed fruits explained 67.90% of the total variance where PC1 accounted for 37.12% and PC2 for 15.99% (Figure 1B). That statistical analyses confirmed that the cvs. “Smoky” and “Thiessen” were characterized by a high content of eleven free amino acids, monophosphate nucleotides as AMP, IMP, GMP, the highest antioxidant activity, and were accountable above all for the strong inhibition of activities of α-glucosidase, α-amylase, and pancreatic lipase enzymes as well as for the strong inhibition of *E. hirae* growth (anti-bacterial activity). “Clones type N” and cv. “Pembina” had a relatively high content of free amino acids as hydroxy-L-proline, urea, leucine, alanine, valine, tyrosine, tryptophan, phenyloalanine, and uridine-5’-monophosphate, while the cvs. ”Martin”, “clone type S” and “clone no 5/6” had low contents of these compounds and exhibited low antioxidant and anti-diabetic activities. Moreover, the clones had a high content of monophosphate nucleotides as XMP, TMP, CMP, and sum of monophosphate nucleotides, as well as free amino acids as threonine, L-citruline, and *O*-phosphoethanolamine.

## 4. Conclusions

This work demonstrated significant differences in both the number of phytochemical components and health-promoting properties of seven selected fruit and fruit fractions in *Amelanchier alnifolia*. These differences depended strongly on the genotype and the fruit fractions (peel, flesh, and seeds). The Canadian cvs. “Smoky” and “Thiessen” are the most valuable in terms of contents of bioactive compounds, high inhibition of hyperglycemia, obesity, and antibacterial activity (*Enterococcus hirae*). The Polish clones (“types N” and “types S”) were characterized by high content of free amino acids and polyphenolic compounds as well as exhibiting antibacterial properties. The peel and seeds of the fruit of the seven tested genotypes were more abundant in bioactive components with high antioxidative than the fruit flesh. The seeds and peel fruit extracts were the most effective α-glucosidase, α-amylase, and pancreatic lipase inhibitors. An assessment of differences in contents of the bioactive components among the fruit fractions may be a key aspect in the development of new food products with distinctive health benefits and sensory qualities. The proper management of by-products (fruit peel and seeds) will bring not only economic but also health benefits. Furthermore, fruit and fruit fractions can be valuable sources of bioactive compounds for the pharmaceutical industry in the production of dietary supplements to support the prevention and treatment of chronic non-communicable diseases.

## Figures and Tables

**Table 1:** The CIE L*a*b* parameters and normalized anthocyanin index (NAI) parameter in the fruit and their fractions of the seven Saskatoon berry genotypes.

Fruit Fraction	Genotypes	Parameters of Color			NAI
		L*	a*	b*	
Fruit	Thiessen	32.71 ± 0.03 ^1^	33.31 ± 0.03	23.54 ± 0.02	0.70 ± 0.00
	Smoky	39.74 ± 0.04	34.55 ± 0.03	24.03 ± 0.02	0.71 ± 0.00
	Martin	42.71 ± 0.04	24.25 ± 0.02	23.83 ± 0.02	0.64 ± 0.00
	Pembina	33.47 ± 0.03	25.22 ± 0.02	18.10 ± 0.02	0.61 ± 0.00
	Clone no 5/6	49.31 ± 0.04	18.33 ± 0.02	24.06 ± 0.02	0.65 ± 0.00
	Clone type S	45.76 ± 0.04	21.92 ± 0.02	21.98 ± 0.02	0.65 ± 0.00
	Clone type N	39.01 ± 0.04	23.53 ± 0.02	19.71 ± 0.02	0.57 ± 0.00
Flesh	Thiessen	55.54 ± 0.05	9.67 ± 0.01	30.73 ± 0.03	0.57 ± 0.00
	Smoky	51.41 ± 0.05	17.62 ± 0.02	40.74 ± 0.04	0.58 ± 0.00
	Martin	55.26 ± 0.05	12.37 ± 0.01	40.41 ± 0.04	0.52 ± 0.00
	Pembina	43.30 ± 0.04	12.86 ± 0.01	30.69 ± 0.03	0.50 ± 0.00
	Clone no 5/6	63.80 ± 0.06	9.35 ± 0.01	40.80 ± 0.04	0.53 ± 0.00
	Clone type S	59.20 ± 0.05	11.18 ± 0.01	37.27 ± 0.03	0.54 ± 0.00
	Clone type N	50.47 ± 0.05	12.00 ± 0.01	33.42 ± 0.03	0.62 ± 0.00
Peel	Thiessen	19.07 ± 0.02	46.36 ± 0.04	26.85 ± 0.02	0.83 ± 0.00
	Smoky	23.17 ± 0.02	48.09 ± 0.04	29.82 ± 0.03	0.85 ± 0.00
	Martin	24.90 ± 0.02	33.75 ± 0.03	29.57 ± 0.03	0.76 ± 0.00
	Pembina	19.51 ± 0.02	35.10 ± 0.03	22.46 ± 0.02	0.73 ± 0.00
	Clone no 5/6	28.75 ± 0.03	25.51 ± 0.02	29.86 ± 0.03	0.77 ± 0.00
	Clone type S	26.68 ± 0.02	30.51 ± 0.03	27.28 ± 0.02	0.78 ± 0.00
	Clone type N	23.38 ± 0.02	48.07 ± 0.04	24.46 ± 0.02	0.83 ± 0.00
Seeds	Thiessen	51.26 ± 0.05	11.88 ± 0.01	20.96 ± 0.02	0.00 ± 0.00
	Smoky	56.70 ± 0.05	12.72 ± 0.01	26.43 ± 0.02	0.00 ± 0.00
	Martin	60.94 ± 0.05	8.92 ± 0.01	26.21 ± 0.02	0.00 ± 0.00
	Pembina	47.76 ± 0.04	9.28 ± 0.01	19.91 ± 0.02	0.00 ± 0.00
	Clone no 5/6	70.36 ± 0.06	6.75 ± 0.01	26.46 ± 0.02	0.00 ± 0.00
	Clone type S	65.29 ± 0.06	8.07 ± 0.01	24.18 ± 0.02	0.00 ± 0.00
	Clone type N	55.66 ± 0.05	8.66 ± 0.01	21.68 ± 0.02	0.00 ± 0.00
Fruit ^3^		40.39 ^c2^	25.87 ^b^	22.18 ^d^	0.65 ^b^
Flesh		54.14 ^b^	12.15 ^c^	36.29 ^a^	0.55 ^c^
Peel		23.64 ^d^	38.20 ^a^	27.19 ^b^	0.79 ^a^
Seeds		58.28 ^a^	9.47 ^d^	23.69 ^c^	0.00 ^d^

^1^ Values are means ± standard deviation. *n* = 3; ^2^ different letters represent significant differences (*p* < 0.05); ^3^ mean results.

**Table 2:** The content of monophosphate nucleotides in the fruit and their fractions of the tested berry fruits (mg/100 g d.w.).

Fruit Fraction	Genotypes	Monophosphate Nucleotides *							
		IMP	UMP	CMP	GMP	XMP	TMP	AMP	SUM
Fruit	Thiessen	1.04 ± 0.01 ^1^	5.13 ± 0.04	9.04 ± 0.07	1.25 ± 0.01	10.66 ± 0.09	1.74 ± 0.01	1.36 ± 0.01	30.22 ± 0.24
	Smoky	1.06 ± 0.01	4.09 ± 0.03	10.69 ± 0.09	0.72 ± 0.01	13.39 ± 0.11	1.16 ± 0.01	0.98 ± 0.01	32.09 ± 0.26
	Martin	1.25 ± 0.01	4.68 ± 0.04	10.81 ± 0.09	1.66 ± 0.01	12.42 ± 0.10	1.33 ± 0.01	1.64 ± 0.01	33.80 ± 0.27
	Pembina	0.55 ± 0.00	4.14 ± 0.03	4.10 ± 0.03	0.52 ± 0.00	4.81 ± 0.04	0.69 ± 0.01	0.74 ± 0.01	15.55 ± 0.12
	Clone no 5/6	1.07 ± 0.01	4.90 ± 0.04	10.91 ± 0.09	1.28 ± 0.01	12.31 ± 0.10	1.57 ± 0.01	1.23 ± 0.01	33.27 ± 0.27
	Clone type S	0.53 ± 0.00	2.53 ± 0.02	8.88 ± 0.07	0.50 ± 0.00	10.12 ± 0.08	0.73 ± 0.01	0.77 ± 0.01	24.06 ± 0.19
	Clone type N	0.77 ± 0.01	2.93 ± 0.02	7.58 ± 0.06	0.85 ± 0.01	9.03 ± 0.07	0.83 ± 0.01	0.92 ± 0.01	22.92 ± 0.18
Flesh	Thiessen	0.57 ± 0.00	6.03 ± 0.05	7.68 ± 0.06	0.97 ± 0.01	8.59 ± 0.07	0.92 ± 0.01	1.18 ± 0.01	25.93 ± 0.21
	Smoky	0.89 ± 0.01	4.39 ± 0.04	10.69 ± 0.09	1.34 ± 0.01	10.92 ± 0.1	1.73 ± 0.01	1.00 ± 0.01	30.96 ± 0.26
	Martin	1.02 ± 0.01	3.80 ± 0.01	3.18 ± 0.01	0.57 ± 0.00	2.26 ± 0.01	0.67 ± 0.01	0.72 ± 0.01	12.22 ± 0.05
	Pembina	1.07 ± 0.01	4.01 ± 0.03	2.78 ± 0.10	1.77 ± 0.01	3.89 ± 0.11	1.33 ± 0.01	1.20 ± 0.01	16.05 ± 0.29
	Clone no 5/6	0.47 ± 0.00	3.55 ± 0.03	4.85 ± 0.04	0.55 ± 0.00	5.38 ± 0.04	0.69 ± 0.01	0.54 ± 0.00	16.03 ± 0.13
	Clone type S	1.04 ± 0.01	5.06 ± 0.04	11.76 ± 0.09	1.27 ± 0.01	7.08 ± 0.10	1.59 ± 0.01	0.95 ± 0.01	28.75 ± 0.28
	Clone type N	0.50 ± 0.00	4.52 ± 0.00	7.81 ± 0.01	0.39 ± 0.00	0.82 ± 0.01	0.77 ± 0.00	0.63 ± 0.01	15.49 ± 0.03
Peel	Thiessen	0.76 ± 0.01	3.03 ± 0.02	8.17 ± 0.07	0.84 ± 0.01	9.59 ± 0.08	0.84 ± 0.01	0.72 ± 0.01	23.94 ± 0.19
	Smoky	0.56 ± 0.00	6.22 ± 0.05	8.27 ± 0.07	0.96 ± 0.01	9.12 ± 0.07	0.93 ± 0.01	0.91 ± 0.01	26.98 ± 0.22
	Martin	0.97 ± 0.01	4.80 ± 0.04	11.21 ± 0.09	1.17 ± 0.01	12.53 ± 0.1	2.81 ± 0.02	1.21 ± 0.01	34.70 ± 0.28
	Pembina	1.02 ± 0.01	2.80 ± 0.01	5.18 ± 0.01	0.57 ± 0.00	7.26 ± 0.01	0.67 ± 0.01	0.72 ± 0.01	18.22 ± 0.05
	Clone no 5/6	1.17 ± 0.01	4.38 ± 0.04	13.39 ± 0.11	1.55 ± 0.01	14.59 ± 0.12	2.16 ± 0.02	1.46 ± 0.01	38.71 ± 0.31
	Clone type S	0.52 ± 0.00	3.88 ± 0.03	5.09 ± 0.04	0.48 ± 0.00	5.65 ± 0.05	1.11 ± 0.01	0.65 ± 0.01	17.38 ± 0.14
	Clone type N	0.83 ± 0.01	4.78 ± 0.04	10.99 ± 0.09	1.03 ± 0.01	12.02 ± 0.1	1.29 ± 0.01	0.52 ± 0.00	31.45 ± 0.25
Seeds	Thiessen	1.02 ± 0.01	4.80 ± 0.01	10.18 ± 0.01	0.57 ± 0.00	9.26 ± 0.01	0.67 ± 0.01	0.72 ± 0.01	33.44 ± 0.05
	Smoky	1.00 ± 0.01	4.36 ± 0.03	13.13 ± 0.11	1.37 ± 0.01	14.01 ± 0.11	0.99 ± 0.01	0.62 ± 0.00	35.47 ± 0.28
	Martin	0.44 ± 0.00	3.86 ± 0.03	4.99 ± 0.04	0.43 ± 0.00	5.42 ± 0.04	0.51 ± 0.00	0.28 ± 0.00	15.92 ± 0.13
	Pembina	0.95 ± 0.01	0.00 ± 0.00	9.29 ± 0.07	1.18 ± 0.01	10.66 ± 0.09	1.98 ± 0.02	0.52 ± 0.00	24.59 ± 0.20
	Clone no 5/6	0.47 ± 0.00	6.62 ± 0.05	6.71 ± 0.05	0.63 ± 0.01	7.66 ± 0.06	1.39 ± 0.01	0.54 ± 0.00	24.02 ± 0.19
	Clone type S	0.84 ± 0.01	3.56 ± 0.03	6.93 ± 0.06	1.08 ± 0.01	8.56 ± 0.07	1.31 ± 0.01	0.49 ± 0.00	22.77 ± 0.18
	Clone type N	0.77 ± 0.01	5.59 ± 0.04	3.86 ± 0.03	1.15 ± 0.01	4.26 ± 0.03	0.69 ± 0.01	1.46 ± 0.01	17.77 ± 0.14
Fruit ^3^		0.97 ^b2^	4.15 ^b^	10.56 ^a^	1.22 ^b^	11.88 ^a^	1.81 ^a^	0.97 ^b^	31.56 ^a^
Flesh		0.80 ^c^	2.31 ^d^	4.38 ^d^	0.57 ^d^	5.11 ^d^	0.82 ^c^	0.73 ^d^	14.71 ^d^
Peel		0.98 ^a^	3.85 ^c^	10.40 ^b^	1.30 ^a^	11.73 ^b^	1.26 ^b^	1.01 ^a^	30.52 ^b^
Seeds		0.56 ^d^	4.75 ^a^	5.55 ^c^	0.72 ^c^	6.18 ^c^	0.79 ^d^	0.82 ^c^	19.37 ^c^

^1^ Values are means ± standard deviation. *n* = 3; ^2^ different letters represent significant differences (*p* < 0.05); ^3^ mean results. Explanations: * selected monophosphate nucleotides: IMP, inosine 5′-monophosphate; UMP, uridine 5’-monophosphate; CMP, cytidine 5’-monophosphate; GMP, guanosine 5’-monophosphate; XMP, xanthosine 5′-monophosphate; TMP, thymidine 5’-monophosphate; AMP, adenosine 5’-monophosphate.

**Table 3:** The content of essential free amino acids in the fruit and their components fractions of the seven berry genotypes (mg/100 g d.w.).

Fruit Fraction	Genotypes	Arg	His	Met	Lys	Leu	Thr	Val	Phe	PE	Trp	Ur	3MH
Fruit	Thiessen	8.10 ± 0.06 ^1^	0.63 ± 0.01	0.02 ± 0.00	0.69 ± 0.01	0.28 ± 0.00	0.01 ± 0.00	0.14 ± 0.00	2.80 ± 0.02	0.65 ± 0.01	0.32 ± 0.00	0.07 ± 0.00	0.12 ± 0.00
	Smoky	3.25 ± 0.03	0.99 ± 0.01	0.02 ± 0.00	0.32 ± 0.00	0.09 ± 0.00	0.01 ± 0.00	0.01 ± 0.00	3.48 ± 0.03	0.85 ± 0.01	0.33 ± 0.00	0.07 ± 0.00	0.08 ± 0.00
	Martin	8.65 ± 0.07	1.23 ± 0.01	0.04 ± 0.00	0.66 ± 0.01	0.44 ± 0.00	0.01 ± 0.00	0.29 ± 0.00	3.95 ± 0.03	0.57 ± 0.00	0.24 ± 0.00	0.14 ± 0.00	0.04 ± 0.00
	Pembina	9.15 ± 0.15	2.61 ± 0.02	0.11 ± 0.00	0.90 ± 0.01	0.57 ± 0.00	0.01 ± 0.00	0.37 ± 0.00	6.24 ± 0.05	0.43 ± 0.00	3.88 ± 0.03	0.07 ± 0.00	0.57 ± 0.00
	Clone no 5/6	8.66 ± 0.07	2.23 ± 0.02	0.02 ± 0.00	0.57 ± 0.00	0.23 ± 0.00	0.01 ± 0.00	0.22 ± 0.00	3.38 ± 0.03	0.73 ± 0.01	0.56 ± 0.00	0.07 ± 0.00	0.28 ± 0.00
	Clone type S	5.92 ± 0.01	0.60 ± 0.00	0.02 ± 0.00	0.20 ± 0.00	0.15 ± 0.00	0.01 ± 0.00	0.13 ± 0.00	1.85 ± 0.01	0.64 ± 0.01	0.11 ± 0.00	0.10 ± 0.00	0.02 ± 0.00
	Clone type N	5.19 ± 0.01	0.25 ± 0.00	0.02 ± 0.00	0.26 ± 0.00	0.14 ± 0.00	0.01 ± 0.00	0.11 ± 0.00	1.83 ± 0.01	0.55 ± 0.00	0.15 ± 0.00	0.07 ± 0.00	0.03 ± 0.00
Flesh	Thiessen	12.52 ± 0.1	5.29 ± 0.01	0.10 ± 0.00	1.35 ± 0.01	0.64 ± 0.01	0.02 ± 0.00	0.36 ± 0.00	9.35 ± 0.07	0.33 ± 0.00	1.11 ± 0.01	0.07 ± 0.00	0.22 ± 0.00
	Smoky	4.66 ± 0.04	3.31 ± 0.03	0.02 ± 0.00	0.47 ± 0.00	0.34 ± 0.00	0.01 ± 0.00	0.33 ± 0.00	4.52 ± 0.04	0.60 ± 0.00	1.06 ± 0.01	0.07 ± 0.00	0.36 ± 0.00
	Martin	2.87 ± 0.01	5.25 ± 0.04	0.02 ± 0.00	0.22 ± 0.00	0.11 ± 0.00	0.01 ± 0.00	0.12 ± 0.00	5.61 ± 0.04	0.79 ± 0.01	1.09 ± 0.01	0.07 ± 0.00	0.23 ± 0.00
	Pembina	4.98 ± 0.04	6.49 ± 0.05	0.03 ± 0.00	0.45 ± 0.00	0.54 ± 0.00	0.01 ± 0.00	0.71 ± 0.01	6.38 ± 0.05	0.53 ± 0.00	0.8 ± 0.01	0.13 ± 0.00	0.13 ± 0.00
	Clone no 5/6	3.02 ± 0.09	4.82 ± 0.11	0.10 ± 0.00	0.62 ± 0.00	0.70 ± 0.01	0.01 ± 0.00	0.61 ± 0.01	3.61 ± 0.03	0.39 ± 0.00	1.76 ± 0.01	0.07 ± 0.00	1.70 ± 0.01
	Clone type S	5.40 ± 0.04	3.28 ± 0.01	0.03 ± 0.00	0.38 ± 0.00	0.46 ± 0.00	0.01 ± 0.00	0.27 ± 0.00	5.49 ± 0.04	0.53 ± 0.00	1.67 ± 0.01	0.08 ± 0.00	0.10 ± 0.00
	Clone type N	9.17 ± 0.02	4.03 ± 0.02	0.03 ± 0.00	0.18 ± 0.00	0.15 ± 0.00	0.01 ± 0.00	0.22 ± 0.00	6.81 ± 0.05	0.69 ± 0.01	1.73 ± 0.01	0.08 ± 0.00	0.06 ± 0.00
Peel	Thiessen	8.76 ± 0.05	2.51 ± 0.02	0.05 ± 0.00	0.36 ± 0.00	0.73 ± 0.01	0.02 ± 0.00	0.58 ± 0.00	7.75 ± 0.06	0.47 ± 0.00	1.27 ± 0.01	0.15 ± 0.00	0.04 ± 0.00
	Smoky	9.75 ± 0.10	4.34 ± 0.04	0.05 ± 0.00	0.49 ± 0.00	0.94 ± 0.01	0.02 ± 0.00	0.75 ± 0.01	4.38 ± 0.04	0.35 ± 0.00	2.78 ± 0.02	0.08 ± 0.00	0.47 ± 0.00
	Martin	7.05 ± 0.05	1.05 ± 0.01	0.02 ± 0.00	0.26 ± 0.00	0.34 ± 0.00	0.02 ± 0.00	0.18 ± 0.00	4.14 ± 0.03	0.68 ± 0.01	0.80 ± 0.01	0.07 ± 0.00	0.11 ± 0.00
	Pembina	4.43 ± 0.02	2.67 ± 0.01	0.02 ± 0.00	0.12 ± 0.00	0.11 ± 0.00	0.01 ± 0.00	0.10 ± 0.00	5.14 ± 0.04	0.90 ± 0.01	0.83 ± 0.01	0.07 ± 0.00	0.07 ± 0.00
	Clone no 5/6	6.46 ± 0.05	2.06 ± 0.02	0.04 ± 0.00	0.25 ± 0.00	0.54 ± 0.00	0.02 ± 0.00	0.38 ± 0.00	5.84 ± 0.05	0.60 ± 0.00	0.61 ± 0.00	0.13 ± 0.00	0.04 ± 0.00
	Clone type S	9.29 ± 0.11	4.39 ± 0.04	0.10 ± 0.00	0.34 ± 0.00	0.69 ± 0.01	0.02 ± 0.00	0.49 ± 0.00	9.23 ± 0.07	0.45 ± 0.00	9.77 ± 0.08	0.07 ± 0.00	0.51 ± 0.00
	Clone type N	8.08 ± 0.06	0.86 ± 0.01	0.02 ± 0.00	0.38 ± 0.00	0.17 ± 0.00	0.02 ± 0.00	0.16 ± 0.00	3.47 ± 0.03	0.57 ± 0.00	0.65 ± 0.01	0.07 ± 0.00	0.14 ± 0.00
Seeds	Thiessen	8.84 ± 0.02	1.36 ± 0.01	0.02 ± 0.00	1.18 ± 0.00	0.06 ± 0.00	0.02 ± 0.00	0.10 ± 0.00	4.31 ± 0.03	0.75 ± 0.01	1.68 ± 0.01	0.07 ± 0.00	0.09 ± 0.00
	Smoky	7.56 ± 0.06	1.69 ± 0.01	0.03 ± 0.00	0.36 ± 0.00	0.27 ± 0.00	0.02 ± 0.00	0.34 ± 0.00	4.90 ± 0.04	0.51 ± 0.00	1.50 ± 0.00	0.13 ± 0.00	0.05 ± 0.00
	Martin	7.56 ± 0.06	1.69 ± 0.01	0.03 ± 0.00	0.36 ± 0.00	0.27 ± 0.00	0.02 ± 0.00	0.34 ± 0.00	4.90 ± 0.04	0.51 ± 0.00	0.50 ± 0.00	0.13 ± 0.00	0.05 ± 0.00
	Pembina	7.19 ± 0.07	1.81 ± 0.01	0.03 ± 0.00	0.38 ± 0.00	0.48 ± 0.00	0.01 ± 0.00	0.35 ± 0.00	6.27 ± 0.05	0.52 ± 0.00	1.88 ± 0.02	0.07 ± 0.00	0.16 ± 0.00
	Clone no 5/6	3.40 ± 0.01	0.19 ± 0.00	0.02 ± 0.00	0.22 ± 0.00	0.20 ± 0.00	0.01 ± 0.00	0.14 ± 0.00	1.80 ± 0.01	0.33 ± 0.00	0.33 ± 0.00	0.08 ± 0.00	0.02 ± 0.00
	Clone type S	5.29 ± 0.01	0.12 ± 0.00	0.02 ± 0.00	0.22 ± 0.00	0.26 ± 0.00	0.01 ± 0.00	0.13 ± 0.00	1.76 ± 0.01	0.32 ± 0.00	0.31 ± 0.00	0.07 ± 0.00	0.03 ± 0.00
	Clone type N	8.82 ± 0.17	1.50 ± 0.01	0.17 ± 0.00	1.25 ± 0.02	1.00 ± 0.01	0.02 ± 0.00	0.68 ± 0.01	5.00 ± 0.04	0.42 ± 0.00	1.12 ± 0.02	0.07 ± 0.00	0.18 ± 0.00
Fruit ^3^		7.02 ^b2^	1.59 ^d^	0.02 ^c^	0.45 ^b^	0.33 ^c^	0.01 ^a^	0.23 ^c^	4.30 ^c^	0.61 ^b^	0.99 ^b^	0.07 ^d^	0.18 ^b^
Flesh		2.13 ^d^	1.64 ^c^	0.02 ^c^	0.20 ^d^	0.12 ^d^	0.01 ^a^	0.05 ^d^	4.14 ^d^	0.71 ^a^	0.73 ^c^	0.08 ^c^	0.08 ^c^
Peel		5.13 ^c^	2.05 ^b^	0.03 ^b^	0.37 ^c^	0.42 ^b^	0.02 ^a^	0.36 ^b^	4.63 ^b^	0.51 ^c^	0.55 ^d^	0.12 ^a^	0.05 ^d^
Seeds		7.95 ^a^	4.38 ^a^	0.11 ^a^	0.90 ^a^	0.69 ^a^	0.02 ^a^	0.56 ^a^	6.10 ^a^	0.41 ^d^	1.04 ^a^	0.08 ^b^	0.53 ^a^

^1^ Values are means ± standard deviation. *n* = 3; ^2^ different letters represent significant differences (*p* < 0.05); ^3^ mean results. Explanations: Arg, arginine; His, histidine, Met, methionine; Lys, lysine; Leu, leucine; Thr, threonine; Val, valine; Phe, phenyloalanine; PE, *O*-phosphoethanolamine; Try; tryptophan; Ur, urea; 3MH, 3 methyl-L-histidine.

**Table 4:** The content of non-essential free amino acids in the fruit and their components fractions of the seven berry genotypes (mg/100 g d.w.).

Fruit Fraction	Genotypes	Glu	Asp	Pro	Hpro	Ser	Gly	Ala	Tyr	Cit	Ort	Asn	Arg
Fruit	Thiessen	5.62 ± 0.04	13.37 ± 0.11	1.37 ± 0.01	0.00 ± 0.00	4.26 ± 0.03	0.03 ± 0.00	0.18 ± 0.00	0.36 ± 0.00	0.05 ± 0.00	0.84 ± 0.01	10.38 ± 0.12	5.62 ± 0.04
	Smoky	3.88 ± 0.03	13.89 ± 0.11	1.48 ± 0.00	0.02 ± 0.00	3.51 ± 0.03	0.04 ± 0.00	0.11 ± 0.00	0.21 ± 0.00	0.08 ± 0.00	0.51 ± 0.00	10.73 ± 0.09	3.88 ± 0.03
	Martin	4.87 ± 0.04	9.80 ± 0.08	1.34 ± 0.01	0.02 ± 0.00	3.21 ± 0.03	0.04 ± 0.00	0.21 ± 0.00	0.66 ± 0.01	0.08 ± 0.00	1.93 ± 0.05	8.77 ± 0.05	4.87 ± 0.04
	Pembina	6.32 ± 0.05	8.86 ± 0.07	1.61 ± 0.01	0.03 ± 0.00	3.67 ± 0.03	0.06 ± 0.00	0.32 ± 0.00	1.09 ± 0.01	0.05 ± 0.00	0.60 ± 0.00	12.40 ± 0.11	6.32 ± 0.05
	Clone no 5/6	4.95 ± 0.04	10.17 ± 0.08	1.79 ± 0.01	0.03 ± 0.00	4.17 ± 0.03	0.00 ± 0.00	0.12 ± 0.00	0.35 ± 0.00	0.05 ± 0.00	0.78 ± 0.01	11.46 ± 0.11	4.95 ± 0.04
	Clone type S	2.04 ± 0.02	9.15 ± 0.07	0.89 ± 0.01	0.02 ± 0.00	3.47 ± 0.03	0.03 ± 0.00	0.15 ± 0.00	0.11 ± 0.00	0.08 ± 0.00	0.14 ± 0.00	9.76 ± 0.07	2.04 ± 0.02
	Clone type N	2.30 ± 0.02	7.20 ± 0.06	1.04 ± 0.01	0.03 ± 0.00	2.67 ± 0.02	0.03 ± 0.00	0.18 ± 0.00	0.13 ± 0.00	0.05 ± 0.00	0.53 ± 0.00	9.46 ± 0.04	2.30 ± 0.02
Flesh	Thiessen	5.56 ± 0.04	6.24 ± 0.05	2.32 ± 0.02	0.03 ± 0.00	4.37 ± 0.03	0.17 ± 0.00	0.52 ± 0.00	0.89 ± 0.01	0.13 ± 0.00	0.62 ± 0.00	14.98 ± 0.12	5.56 ± 0.04
	Smoky	4.81 ± 0.04	13.64 ± 0.11	2.21 ± 0.02	0.00 ± 0.00	3.26 ± 0.03	0.04 ± 0.00	0.19 ± 0.00	0.43 ± 0.00	0.06 ± 0.00	0.60 ± 0.00	21.45 ± 0.17	4.81 ± 0.04
	Martin	3.32 ± 0.03	14.17 ± 0.11	0.77 ± 0.01	0.01 ± 0.00	2.68 ± 0.02	0.05 ± 0.00	0.13 ± 0.00	0.25 ± 0.00	0.10 ± 0.00	0.36 ± 0.00	14.97 ± 0.12	3.32 ± 0.03
	Pembina	4.17 ± 0.03	9.99 ± 0.08	2.17 ± 0.02	0.01 ± 0.00	2.46 ± 0.02	0.05 ± 0.00	0.47 ± 0.00	0.80 ± 0.01	0.10 ± 0.00	1.24 ± 0.03	9.45 ± 0.08	4.17 ± 0.03
	Clone no 5/6	5.41 ± 0.04	9.03 ± 0.07	2.61 ± 0.02	0.01 ± 0.00	2.81 ± 0.02	0.07 ± 0.00	0.72 ± 0.01	1.32 ± 0.01	0.06 ± 0.00	0.43 ± 0.00	18.69 ± 0.15	5.41 ± 0.04
	Clone type S	3.74 ± 0.03	6.47 ± 0.05	0.42 ± 0.00	0.01 ± 0.00	3.07 ± 0.02	0.00 ± 0.00	0.17 ± 0.00	0.68 ± 0.01	0.08 ± 0.00	0.40 ± 0.00	10.45 ± 0.08	3.74 ± 0.03
	Clone type N	2.58 ± 0.02	6.72 ± 0.05	0.15 ± 0.00	0.05 ± 0.00	2.53 ± 0.02	0.01 ± 0.00	0.12 ± 0.00	0.40 ± 0.00	0.13 ± 0.00	0.24 ± 0.00	7.29 ± 0.06	2.58 ± 0.02
Peel	Thiessen	3.23 ± 0.03	4.74 ± 0.04	0.42 ± 0.00	0.05 ± 0.00	2.31 ± 0.02	0.01 ± 0.00	0.42 ± 0.00	1.27 ± 0.01	0.13 ± 0.00	2.81 ± 0.02	4.60 ± 0.04	3.23 ± 0.03
	Smoky	4.20 ± 0.03	4.28 ± 0.03	0.50 ± 0.00	0.07 ± 0.00	2.64 ± 0.02	0.01 ± 0.00	0.64 ± 0.01	2.09 ± 0.02	0.08 ± 0.00	0.28 ± 0.00	9.11 ± 0.07	4.20 ± 0.03
	Martin	3.02 ± 0.02	5.22 ± 0.04	2.98 ± 0.02	0.01 ± 0.00	3.10 ± 0.02	0.00 ± 0.00	0.25 ± 0.00	0.49 ± 0.00	0.08 ± 0.00	0.19 ± 0.00	5.67 ± 0.05	3.02 ± 0.02
	Pembina	2.08 ± 0.02	5.43 ± 0.04	1.04 ± 0.01	0.03 ± 0.00	2.56 ± 0.02	0.01 ± 0.00	0.04 ± 0.00	0.29 ± 0.00	0.13 ± 0.00	0.11 ± 0.00	3.95 ± 0.03	2.08 ± 0.02
	Clone no 5/6	2.61 ± 0.02	3.83 ± 0.03	2.93 ± 0.02	0.03 ± 0.00	2.34 ± 0.02	0.01 ± 0.00	0.62 ± 0.00	0.90 ± 0.01	0.13 ± 0.00	1.32 ± 0.01	2.50 ± 0.02	2.61 ± 0.02
	Clone type S	3.39 ± 0.03	3.46 ± 0.03	3.51 ± 0.03	0.04 ± 0.00	2.68 ± 0.02	0.01 ± 0.00	0.96 ± 0.01	1.49 ± 0.01	0.08 ± 0.00	0.13 ± 0.00	4.94 ± 0.04	3.39 ± 0.03
	Clone type N	2.36 ± 0.02	6.56 ± 0.05	0.90 ± 0.01	0.01 ± 0.00	1.91 ± 0.02	0.03 ± 0.00	0.11 ± 0.00	0.19 ± 0.00	0.07 ± 0.00	0.22 ± 0.00	12.84 ± 0.1	2.36 ± 0.02
Seeds	Thiessen	4.63 ± 0.01	6.81 ± 0.05	0.32 ± 0.00	0.03 ± 0.00	1.58 ± 0.01	0.04 ± 0.00	0.12 ± 0.00	0.11 ± 0.00	0.11 ± 0.00	0.13 ± 0.00	8.96 ± 0.07	4.63 ± 0.01
	Smoky	2.04 ± 0.02	4.81 ± 0.04	0.89 ± 0.01	0.03 ± 0.00	1.44 ± 0.01	0.04 ± 0.00	0.28 ± 0.00	0.35 ± 0.00	0.11 ± 0.00	1.56 ± 0.01	5.65 ± 0.05	2.04 ± 0.02
	Martin	2.04 ± 0.02	4.81 ± 0.04	0.89 ± 0.01	0.03 ± 0.00	1.44 ± 0.01	0.04 ± 0.00	0.28 ± 0.00	0.35 ± 0.00	0.11 ± 0.00	1.56 ± 0.01	5.65 ± 0.05	2.04 ± 0.02
	Pembina	2.64 ± 0.03	4.86 ± 0.04	0.68 ± 0.01	0.02 ± 0.00	2.19 ± 0.02	0.02 ± 0.00	0.26 ± 0.00	0.61 ± 0.00	0.07 ± 0.00	0.21 ± 0.00	7.23 ± 0.06	3.64 ± 0.03
	Clone no 5/6	2.15 ± 0.02	3.37 ± 0.03	0.31 ± 0.00	0.03 ± 0.00	1.56 ± 0.01	0.04 ± 0.00	0.28 ± 0.00	0.27 ± 0.00	0.08 ± 0.00	0.09 ± 0.00	4.85 ± 0.04	2.15 ± 0.02
	Clone type S	2.24 ± 0.02	3.41 ± 0.03	0.27 ± 0.00	0.03 ± 0.00	1.18 ± 0.01	0.04 ± 0.00	0.32 ± 0.00	0.22 ± 0.00	0.08 ± 0.00	0.09 ± 0.00	6.85 ± 0.03	2.24 ± 0.02
	Clone type N	6.05 ± 0.06	5.26 ± 0.04	0.97 ± 0.01	0.03 ± 0.00	2.62 ± 0.02	0.72 ± 0.01	0.75 ± 0.01	1.83 ± 0.01	0.01 ± 0.00	0.38 ± 0.00	9.33 ± 0.10	5.05 ± 0.06
Fruit ^3^		4.02 ^b2^	8.61 ^a^	1.48 ^b^	0.01 ^c^	3.14 ^a^	0.02 ^b^	0.17 ^c^	0.44 ^c^	0.07 ^b^	0.46 ^c^	9.35 ^a^	4.02 ^b^
Flesh		2.53 ^d^	8.51 ^b^	0.57 ^d^	0.03 ^b^	2.55 ^c^	0.03 ^b^	0.08 ^d^	0.23 ^d^	0.10 ^a^	0.23 ^d^	8.50 ^c^	2.53 ^d^
Peel		3.07 ^c^	6.25 ^c^	1.29 ^c^	0.03 ^b^	2.23 ^d^	0.03 ^b^	0.36 ^b^	0.62 ^b^	0.10 ^a^	2.35 ^a^	5.47 ^d^	3.07 ^c^
Seeds		5.00 ^a^	5.99 ^d^	1.77 ^a^	0.04 ^a^	2.89 ^b^	0.15 ^a^	0.60 ^a^	1.30 ^a^	0.07 ^b^	0.57 ^b^	9.30 ^b^	5.00 ^a^

^1^ Values are means ± standard deviation. *n* = 3; ^2^ different letters represent significant differences (*p* < 0.05); ^3^ mean results. Explanations: Glu, glutamic acid; Asp, aspartic acid; Pro, proline; Hpro, hydroxy-L-proline, Ser, serine; Gly, glycine; Ala, alanine; Tyr, tyrosine; Cit, L-citruline; Ort, L-ornithine; Asn, asparagine.

**Table 5:** The content of total polyphenolic compounds, antioxidant activity, and antidiabetic potential in the fruit and their components fractions of the seven genotypes.

Fruit Fraction	Genotypes	Polyphenols (g GAE/100 g d.w.)	Antioxidative Activity	Antidiabetic Potency (IC_50_ (mg/mL))			Anti-Bacterial Activity [mm]
			ORAC (mmol vit.C/100 g d.w.)	α-Amylase	α-Glucosidase	Pancreatic Lipase	*E. hirae*
**Fruit**	**Thiessen**	2.27 ± 0.02 ^1^	61.02 ± 0.05	24.33 ± 0.02	38.07 ± 0.03	109.03 ± 0.10	5.88 ± 0.24
	Smoky	2.04 ± 0.02	62.05 ± 0.06	31.70 ± 0.03	42.23 ± 0.04	132.63 ± 0.12	5.35 ± 0.01
	Martin	2.03 ± 0.02	40.12 ± 0.04	26.40 ± 0.02	35.30 ± 0.03	90.83 ± 0.08	4.34 ± 0.07
	Pembina	1.43 ± 0.01	35.37 ± 0.03	19.03 ± 0.02	27.83 ± 0.03	88.20 ± 0.08	2.57 ± 0.19
	Clone no 5/6	1.11 ± 0.01	46.43 ± 0.04	23.43 ± 0.02	30.17 ± 0.03	95.10 ± 0.09	2.83 ± 0.19
	Clone type S	1.68 ± 0.01	47.23 ± 0.04	22.73 ± 0.02	29.87 ± 0.03	91.93 ± 0.08	8.03 ± 0.50
	Clone type N	1.98 ± 0.02	49.74 ± 0.04	18.33 ± 0.02	32.83 ± 0.03	81.93 ± 0.07	5.35 ± 0.32
Flesh	Thiessen	0.96 ± 0.01	25.01 ± 0.02	33.20 ± 0.03	39.47 ± 0.04	101.24 ± 0.09	0.00 ± 0.00
	Smoky	0.87 ± 0.01	28.59 ± 0.03	42.15 ± 0.04	43.79 ± 0.04	123.15 ± 0.11	0.00 ± 0.00
	Martin	0.45 ± 0.00	28.36 ± 0.03	36.03 ± 0.03	36.60 ± 0.03	84.34 ± 0.08	0.00 ± 0.00
	Pembina	0.49 ± 0.00	23.68 ± 0.02	25.29 ± 0.02	28.86 ± 0.03	81.90 ± 0.07	0.00 ± 0.00
	Clone no 5/6	0.52 ± 0.00	20.70 ± 0.02	31.98 ± 0.03	31.28 ± 0.03	88.31 ± 0.08	0.00 ± 0.00
	Clone type S	0.75 ± 0.01	26.64 ± 0.02	30.23 ± 0.03	30.97 ± 0.03	85.37 ± 0.08	0.00 ± 0.00
	Clone type N	0.78 ± 0.01	33.72 ± 0.03	22.53 ± 0.02	33.87 ± 0.03	83.97 ± 0.08	0.00 ± 0.00
Peel	Thiessen	2.86 ± 0.02	151.96 ± 0.14	14.13 ± 0.01	33.40 ± 0.03	118.46 ± 0.11	0.00 ± 0.00
	Smoky	2.76 ± 0.02	105.05 ± 0.09	18.41 ± 0.02	37.06 ± 0.03	144.10 ± 0.13	0.00 ± 0.00
	Martin	1.56 ± 0.01	91.94 ± 0.08	15.34 ± 0.01	30.97 ± 0.03	98.69 ± 0.09	0.00 ± 0.00
	Pembina	1.11 ± 0.01	113.31 ± 0.1	11.05 ± 0.01	24.42 ± 0.02	95.83 ± 0.09	0.00 ± 0.00
	Clone no 5/6	1.60 ± 0.01	117.65 ± 0.11	13.61 ± 0.01	26.47 ± 0.02	103.34 ± 0.09	0.00 ± 0.00
	Clone type S	1.91 ± 0.02	142.59 ± 0.13	13.21 ± 0.01	26.21 ± 0.02	99.89 ± 0.09	0.00 ± 0.00
	Clone type N	1.96 ± 0.02	136.03 ± 0.12	12.27 ± 0.01	23.60 ± 0.02	94.50 ± 0.09	0.00 ± 0.00
Seeds	Thiessen	0.77 ± 0.01	14.09 ± 0.01	42.30 ± 0.04	81.90 ± 0.07	0.00 ± 0.00	0.00 ± 0.00
	Smoky	0.53 ± 0.00	9.30 ± 0.01	55.12 ± 0.05	90.87 ± 0.08	0.00 ± 0.00	0.00 ± 0.00
	Martin	0.21 ± 0.00	10.17 ± 0.01	45.90 ± 0.04	75.95 ± 0.07	0.00 ± 0.00	0.00 ± 0.00
	Pembina	0.14 ± 0.00	8.96 ± 0.01	33.08 ± 0.03	59.89 ± 0.05	0.00 ± 0.00	0.00 ± 0.00
	Clone no 5/6	0.10 ± 0.00	12.59 ± 0.01	40.74 ± 0.04	64.90 ± 0.06	0.00 ± 0.00	0.00 ± 0.00
	Clone type S	0.26 ± 0.00	8.99 ± 0.01	39.52 ± 0.04	64.26 ± 0.06	0.00 ± 0.00	0.00 ± 0.00
	Clone type N	0.26 ± 0.00	18.47 ± 0.02	51.07 ± 0.05	77.70 ± 0.07	0.00 ± 0.00	0.00 ± 0.00
Fruit ^3^		1.79 ^b2^	48.85 ^b^	23.71 ^c^	33.76 ^c^	98.52 ^b^	4.91 ^a^
Flesh		0.69	26.67 ^c^	31.63 ^b^	34.97 ^b^	92.61 ^c^	0.00 ^b^
Peel		2.00 ^a^	122.65 ^a^	14.00 ^d^	28.88 ^d^	107.83 ^a^	0.00 ^b^
Seeds		0.32	11.79 ^d^	43.96 ^a^	73.64 ^a^	0.00 ^d^	0.00 ^b^

^1^ Values are means ± standard deviation. *n* = 3; ^2^ different letters represent significant differences (*p* < 0.05); ^3^ mean results.
